# INCREASING MEDICATION SAFETY AWARENESS IN RURAL OLDER ADULTS

**DOI:** 10.1093/geroni/igac059.3109

**Published:** 2022-12-20

**Authors:** Kara Haughtigan, Susan Jones, Eve Main, Elizabeth Groves, Melinda Joyce

**Affiliations:** School of Nursing and Allied Health, Western Kentucky University, Bowling Green, Kentucky, United States; Western Kentucky University, Bowling Green, Kentucky, United States; Western Kentucky University, Bowling Green, Kentucky, United States; Western Kentucky University, Bowling Green, Kentucky, United States; WKHL/Med Center Health Research Foundation, Bowling Green, Kentucky, United States

## Abstract

Compared to urban populations, rural residents have a higher incidence of chronic diseases and poorer health outcomes. Most medications in the United States are consumed by older adults who are more susceptible to adverse drug events due to the presence of multiple chronic conditions and physiologic changes in the body. This community-based project evaluated the impact of an interdisciplinary medication education intervention on medication knowledge and adherence to medications and refills. The study was a quasi-experimental pretest/posttest design with a convenience sample. The project was marketed via a multi-media approach. Most participants reported learning of the event at a senior center. Each participant received a private educational session for their specific medications with a nurse practitioner, pharmacist, or pharmacy resident. Forty-nine older adults participated in the study with 48 (97.9%) completing both the pretest and posttest. The average age of participants was 71.4 years and the average number of medications per participant was 5.4. There was a statistically significant difference in the adherence to medication and refills subscale score (p = .003). There was no statistically significant difference in the medication knowledge subscale (p = .192), however, the scores did trend upward indicating an increase in medication knowledge. Forty-four (89.8%) participants reported they were more comfortable understanding their medication since participating in the program and 48 (98%) reported they would recommend the program to others. The results of this study suggest community-based outreach medication educational programs can increase adherence to medication and refills for older adults residing in rural areas.

